# Wastewater treatment alters microbial colonization of microplastics

**DOI:** 10.1371/journal.pone.0244443

**Published:** 2021-01-06

**Authors:** John J. Kelly, Maxwell G. London, Amanda R. McCormick, Miguel Rojas, John W. Scott, Timothy J. Hoellein

**Affiliations:** 1 Department of Biology, Loyola University Chicago, Chicago, Illinois, United States of America; 2 Illinois Sustainable Technology Center, Prairie Research Institute, Champaign, Illinois, United States of America; University of Notre Dame, UNITED STATES

## Abstract

Microplastics are ubiquitous contaminants in aquatic habitats globally, and wastewater treatment plants (WWTPs) are point sources of microplastics. Within aquatic habitats microplastics are colonized by microbial biofilms, which can include pathogenic taxa and taxa associated with plastic breakdown. Microplastics enter WWTPs in sewage and exit in sludge or effluent, but the role that WWTPs play in establishing or modifying microplastic bacterial assemblages is unknown. We analyzed microplastics and associated biofilms in raw sewage, effluent water, and sludge from two WWTPs. Both plants retained >99% of influent microplastics in sludge, and sludge microplastics showed higher bacterial species richness and higher abundance of taxa associated with bioflocculation (e.g. *Xanthomonas*) than influent microplastics, suggesting that colonization of microplastics within the WWTP may play a role in retention. Microplastics in WWTP effluent included significantly lower abundances of some potentially pathogenic bacterial taxa (e.g. *Campylobacteraceae*) compared to influent microplastics; however, other potentially pathogenic taxa (e.g. *Acinetobacter*) remained abundant on effluent microplastics, and several taxa linked to plastic breakdown (e.g. *Klebsiella*, *Pseudomonas*, and *Sphingomonas*) were significantly more abundant on effluent compared to influent microplastics. These results indicate that diverse bacterial assemblages colonize microplastics within sewage and that WWTPs can play a significant role in modifying the microplastic-associated assemblages, which may affect the fate of microplastics within the WWTPs and the environment.

## Introduction

Microplastic particles are contaminants found in aquatic habitats throughout the world, including marine [1–4] and freshwater ecosystems [5–9]. Aquatic organisms ranging from invertebrates to fish can ingest microplastics [10–12], which can negatively affect their digestive systems and cause exposure to toxic chemicals [13–20]. Consumer products, including personal care products (e.g., soaps, lotions, and cleansers that contain microplastic abrasives) and synthetic textiles (fabrics composed of polymers such as acrylic and polyester), are sources of microplastics to the environment [21–23]. Microplastics from consumer products enter domestic wastewater (sewage) through normal use of these products (e.g. washing with soaps and laundering of textiles) and can enter the environment directly if untreated sewage is released through combined sewer overflows or leaking sewage infrastructure. Municipal wastewater treatment plants (WWTPs) remove the majority of microplastics from sewage [24–27], but microplastics are still present in WWTP effluent and the high flow rates of many WWTPs can release large amounts of microplastics [28, 29]. Therefore WWTPs are point sources of microplastics to aquatic environments [6, 8, 30, 31].

Microplastics in both freshwater [6, 8, 32] and marine environments [33–37] are colonized by microbial biofilms, which are diverse assemblages of microorganisms attached to a surface [38]. Many bacterial taxa are capable of biofilm formation, which is controlled by multiple genetic pathways and often involves the expression of type IV pili and the production of extracellular polysaccharides [39]. Biofilms offer microbes protection from a variety of stressors [40] including antimicrobials [41] and can function as reservoirs of antibiotic resistance in the environment [42]. The formation of biofilms on microplastic can influence particle buoyancy [43–45] and transport [46], and may contribute to microplastic breakdown [33, 36]. Microbial communities on microplastics can include potentially pathogenic bacterial taxa, including *Vibrio*, *Campylobacter*, and *Arcobacter* [6, 33, 47, 48], which may colonize microplastics during its transport in sewers and in the wastewater treatment process. WWTPs are designed to remove pathogens from sewage, but microbial communities on microplastics in rivers downstream of WWTPs included bacterial taxa associated with human gastrointestinal infections [6, 8], suggesting that microplastics may play a role in transporting pathogens through WWTPs by providing a buoyant surface for attachment. In addition, microplastics can enhance transport of wastewater-associated taxa within rivers, with potentially negative implications for organisms and ecosystem processes downstream [32]. Currently the composition of microbial assemblages colonizing microplastics within sewage and the potential effects of wastewater treatment on pathogenic microbes associated with microplastics are unknown. Microplastic bacterial assemblages can also include taxa linked to plastic decomposition [6, 8, 49], which could influence the persistence of microplastics in the environment. WWTPs are known to contain microbes responsible for breakdown of a variety of anthropogenic organic compounds [50] including plasticizers [51], but the role that WWTPs play in establishing or altering microplastic bacterial assemblages is unknown. These knowledge gaps limit our ability to manage WWTPs to limit the release of microplastics and microplastic-associated pathogens.

The goal of the current study was to quantify and characterize microplastics in three critical stages of wastewater treatment, and to provide the first analysis of bacterial assemblages attached to microplastics within WWTPs to determine if the composition of these assemblages changes during transport through domestic WWTPs. We sampled two activated sludge WWTPs in Illinois that are point sources of microplastics to their receiving streams [8]. For each plant we characterized microplastics and associated bacterial assemblages in raw sewage, effluent water, and sludge.

## Materials and methods

### Field sites

Samples were collected from two activated sludge WWTPs in DuPage County, IL, that treat primarily domestic wastewater, the Greene Valley Wastewater Facility in Woodridge, IL and the Bartlett Wastewater Treatment Plant in Bartlett, IL. The Greene Valley Wastewater Facility and the Wastewater Treatment Plant of Bartlett both provided access to their facilities for sample collection. At the time of sampling the Woodridge facility filtered its effluent through a 76 cm sand filter while the Bartlett facility did not use a sand filter, and neither plant disinfected its effluent prior to release. Characteristics of the influent wastewater determined by standard methods [52] were provided by both plants. There was a significant difference in flow for the two plants, with the flow for Woodridge being more than 4 fold higher than Bartlett (Table 1). The influent wastewater for the two plants did not differ significantly in total suspended solids or biochemical oxygen demand, but ammonium-N was significantly higher for Woodridge (Table 1).

**Table 1:** Influent wastewater characteristics.

	Flow		Total Suspended Solids		Biochemical Oxygen Demand		Ammonium-N	
Plant	(m^3^ day^-1^)^1^		(mg L^-1^)^2^		(mg L^-1^)^2^		(mg L^-1^)^2^	
Bartlett	7,314	(± 265)	190	(± 23)	177	(± 25)	20.1	(± 0.3)
Woodridge	30,413	(± 679)	257	(± 10)	189	(± 8)	34.0	(± 3.0)
**p value**^3^	< 0.001		0.058		0.665		0.010	

^1^ Data represent mean values ± standard error for daily measurements (n = 21) from the 3 week period immediately prior to sample collection.

^2^ Data represent mean values ± standard error for weekly measurements (n = 3) from the 3 week period immediately prior to sample collection.

^3^ p value based on comparison of data for the two plants by one-way ANOVA.

### Field sampling

We collected replicate samples (n = 4) of untreated wastewater (raw sewage, 15 L per replicate) and unprocessed sludge (4 L per replicate) from each plant in sterile containers and transported them to the lab on ice. We also collected replicate samples (n = 4) of microplastic from effluent water from each plant using 330 micron drift nets held in the flow for 10 min. Water velocity was measured at the center of each net during each deployment with a flow meter (Marsh-McBirney Flo-Mate model 2000 Portable Flowmeter, Loveland, CO). The volume of water passed through the net for each sampling was calculated based on the measured velocity and the area of the net opening. Effluent microplastic samples were stored in sterile glass bottles and transported to the lab on ice. In the lab all samples were stored in the dark at 4°C. All sampling was conducted between October 10 and November 21, 2014.

### Sample processing

Sample processing began within one day of sample collection. Microplastics were isolated and quantified according to adaptation of a common method [5, 6, 53]. Samples were first run through 4.75 mm and 0.3 mm stacked sieves. Sieves were sterilized with ethanol between samples and sterile DI water was used to wash material through the sieves. For effluent samples, all material from the net was sieved. For raw sewage, we sieved the entire 15 L sample, and for sludge we sieved 150 ml homogenized subsamples. After sieving we removed some microplastic particles for bacterial assemblage analysis. Sterile tweezers were used to collect approximately 0.25 ml of randomly selected microplastic pieces from the 0.3 mm sieve for each sample. The microplastics from each sample were stored in a sterile 2ml microcentrifuge tube at -20°C. We recorded the number and shape of each particle removed. Remaining material retained by the 0.3 mm sieve was stored in glass beakers in a drying oven at 75°C for 48 h to remove excess moisture. We digested samples with 30% hydrogen peroxide (H_2_O_2_) and 0.05 M Fe (II) reagent at 75°C [53] to remove organic material. We added sodium chloride to a final concentration of 6M and placed the solution in a glass funnel for salinity-based separation of buoyant microplastic. Funnels were covered with parafilm and left overnight to allow settling of non-buoyant material. We collected floating microplastics on glass fiber filters (0.7 μm nominal pore size, Whatman, Inc. Piscataway, NJ, USA). Filters were placed in aluminum pans, covered with aluminum foil, and dried at 60°C.

### Microplastic quantification

For each filter every piece of plastic was counted manually using a dissecting microscope. Each of the microplastic particles was categorized as fragment, pellet, foam, film, or fiber. Microplastics that were removed for bacterial community analysis prior to digestion were included in counts. Our analyses also included controls (n = 5) to account for procedural and reagent contamination, which consisted of deionized water placed in sample containers and digested in parallel with our samples. Contamination in controls was low (an average of 4.67 fibers per sample, and no contamination by fragments, foam, pellets, or film) and was accounted by subtracting this value from all samples [6, 8].

### Polymer analysis

Representative samples of each microplastic type from each treatment plant were analyzed by pyrolysis gas chromatography mass spectrometry (py-GCMS; CDS Analytical 5200 pyroprobe and Varian 3800 gas chromatograph). A sample was inserted into a quartz capillary tube with quartz wool plugs, then loaded into the pyroprobe and heated to 750°C for 90 s. GC injection port and transfer line were constant at 325°C (split ratio of 10:1). Restek Rtx-5MS capillary column (30 m x 0.25 mm x 0.25 μm df) with carrier gas helium (flow rate of 2.0 mL min^-1^) was used for separation. The oven increased from 40°C to 325°C (heating rate of 10°C min^-1^) and was held for 20 min at 325°C. GC was connected to Saturn 2000 ion trap mass spectrometer, with heated transfer line (325°C) and ion trap (220°C), which collected all mass to charge ions (m/z) from 35–550. We analyzed blanks between each sample to check for carry-over and none occurred. Pyrograms were generated by averaging the mass spectra over the entire chromatogram, and then we searched the CDS Analytical 2013 pyrolysis library for the best match.

### DNA extraction and sequencing

DNA was extracted from microplastic samples collected from raw sewage, sludge, and effluent using MoBio Power Soil DNA kit. Partial 16S rRNA genes were amplified from DNA samples using primers 515F and 806R, which amplify the V4 hypervariable region [54], and amplification was confirmed by agarose gel electrophoresis. DNA extractions were also run without samples as controls for kit contamination, and no amplification was observed for these kit controls. Equimolar amounts of amplicons from each sample were sequenced in a 2 x 250 bp paired-end format using an Illumina MiSeq [55] by DNA Services Facility, University of Illinois at Chicago. Raw sequence data from this study can be downloaded from National Center for Biotechnology Information (NCBI) Sequence Read Archive (SRA) with accession number PRJNA638613.

### Analysis of DNA sequence data

Sequences were processed using mothur v.1.42.3 [56] following the MiSeq Standard Operating Procedure [57]. Briefly, paired reads were assembled and demultiplexed, and any sequences with ambiguities or homopolymers > 8 bases were removed. Sequences were aligned using the SILVA-compatible alignment database available within mothur. Chimeric sequences were removed using VSEARCH [58]. Sequences were classified using the mothur-formatted version of the RDP training set (v.9) and any unknown (i.e. not identified as bacterial), chloroplast, mitochondrial, archaeal, and eukaryotic sequences were removed. Sequences were clustered into operational taxonomic units (OTUs) based on 97% sequence identity and were also grouped into amplicon sequence variants (ASVs). OTUs were assigned to families and genera by comparison to the RDP training set. In order to avoid biases associated with uneven numbers of sequences across samples, the entire dataset was randomly subsampled to 10,226 sequences per sample. For unidentified OTUs we selected a representative sequence, defined as the sequence with minimum distance to other sequences within the OTU, and compared these representative sequences to the NCBI 16S rRNA database using Megablast.

### Statistical analyses

Microplastic concentrations and relative abundances of microplastic categories (fragment, pellet, foam, film, or fiber) were not normally distributed based on the Shapiro-Wilk test (p<0.001). Therefore the effects of treatment plant (Bartlett and Woodridge) and sample type (sewage, effluent, and sludge) on these data were analyzed by the non-parametric Kruskal-Wallis Test followed by the Dwass-Steel-Chritchlow-Fligner Test for all pairwise comparisons using Systat v13. For bacterial assemblage data, we quantified diversity for each sample based on taxonomic richness (i.e. total number of OTUs observed) and the Shannon index [59]. Richness and diversity data were normally distributed based on the Shapiro-Wilk test (p>0.05) so the effects of treatment plant and sample type on richness and diversity were analyzed by two-way ANOVA and Tukey’s HSD Test using Systat v13. Bacterial assemblages were further compared by calculating dissimilarities for each pair of samples based on theta index [60] for both OTUs and ASVs and visualizing the resulting dissimilarity matrices using non-metric multidimensional scaling (nMDS). Statistical significance of differences in assemblages between sample types based on theta index for both OTUs and ASVs was assessed by analysis of molecular variance (AMOVA) [61], a nonparametric analog of traditional analysis of variance. Effect of sample type on relative abundance of the 25 most abundant bacterial families was assessed by one-way ANOVA with Benjamini-Hochberg correction for false discovery rate [62]. Metastats analysis [63] was used to identify OTUs that were differentially abundant between sewage and effluent samples and between sewage and sludge samples, and ANOVA with Benjamini-Hochberg correction for false discovery rate was used to assess significance of differences in relative abundances of these OTUs between the sample types.

## Results

**Fig 1:**
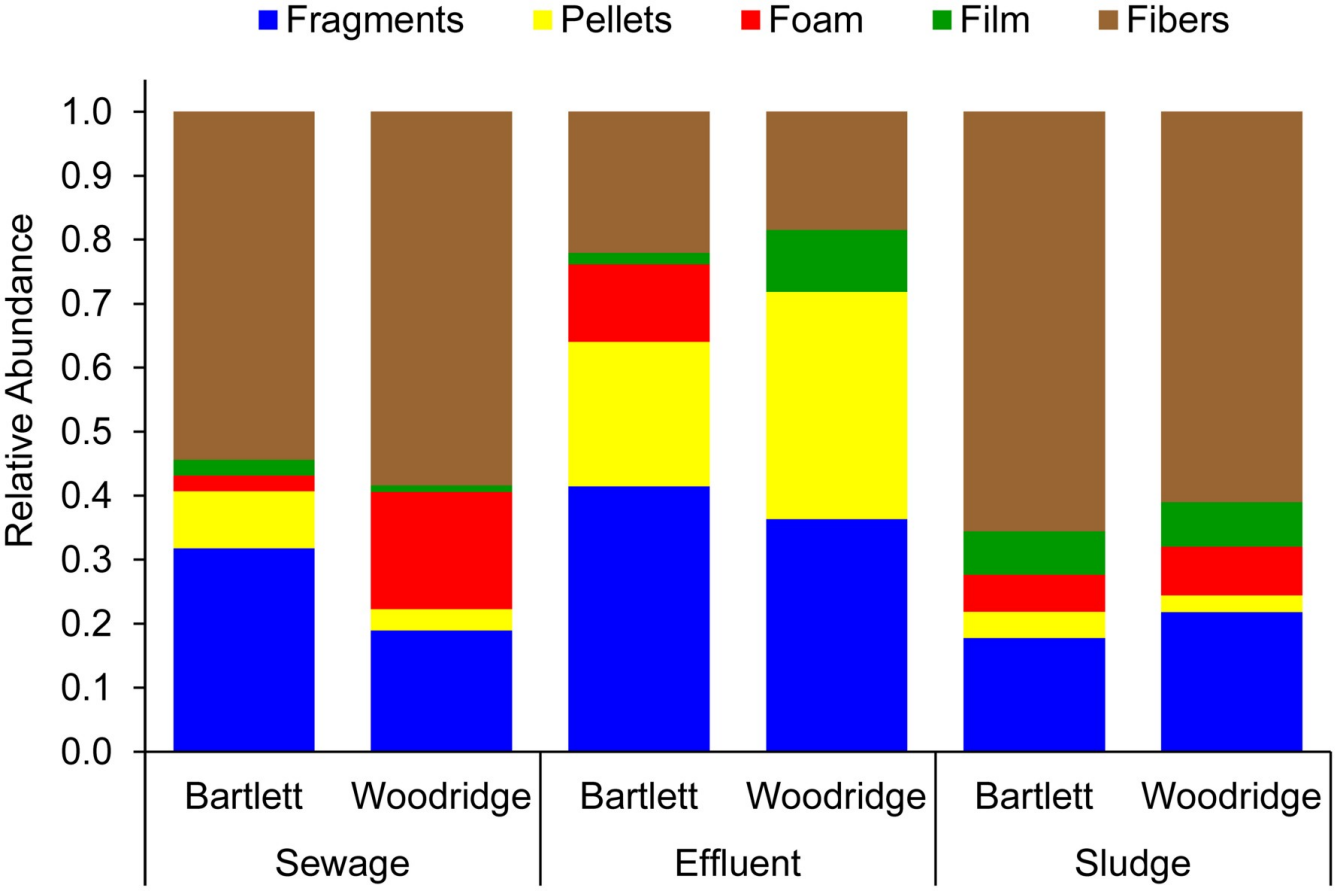
Relative abundance of microplastic particle types within each sample type (sewage, effluent, and sludge) from each of two WWTPs (Bartlett and Woodridge). Each data point represents the mean value (n = 4).

Microplastics were present in all sample types (sewage, effluent, and sludge) from both plants (Bartlett and Woodridge) (Table 2). The microplastics included fragments, pellets, foam, film, and fibers (Fig 1), and were composed of several different polymers, including polyethylene, polypropylene, and polystyrene (S1 Table). There was no significant effect of plant on microplastic concentrations (p = 0.204) but there was a significant effect of sample type (p<0.001) and there were significant differences in microplastic concentrations between each of the sample types (p<0.005 for all pairwise comparisons). When each sample type was compared individually across plants, Bartlett had significantly higher microplastic concentrations than Woodridge in sewage (p = 0.020) and effluent (p = 0.021), but the concentrations were not significantly different between plants for sludge (p = 0.083; Table 2).

**Table 2:** Microplastic concentrations by plant and sample type.

Plant	Sewage^1^		Effluent^1^		Sludge^1^		Retention Rate (%)^2^	Particles Released (#/day)^3^
Bartlett	1,987	(± 68)	1.295	(± 0.214)	335,533	(± 69,761)	99.93	9,471
Woodridge	1,161	(± 178)	0.009	(± 0.001)	140,533	(± 17,294)	99.99	273

^1^ Data represent mean values (n = 4) ± standard error in units of No. m^-3^.

^2^ Retention rate equals the percentage of particles in sewage that were retained by the plant.

^3^ The number of particles released per day was estimated based on the concentration of microplastics in the effluent and the average daily flow of the treatment plant.

Both WWTPs had significantly higher microplastics in the incoming sewage relative to the effluent (p = 0.002), corresponding to a reduction in microplastic concentration of more than 99% for both WWTPs (Table 2). High microplastic retention rates for both WWTPs resulted in an average sludge microplastic concentration > 140,000 pieces/m^3^ (Table 2). While retention of microplastics was high, effluent microplastic concentrations corresponded to releases of approximately 9,470 microplastic pieces per day for Bartlett, and 273 microplastic pieces per day for Woodridge (Table 2).

There were some differences in microplastic particle types among the sample types within the WWTPs. Fibers were the most common microplastic type in incoming sewage for both Woodridge and Bartlett, accounting for > 50% of sewage microplastic for both plants (Fig 1). Relative abundance of fibers showed no difference between plants (p = 0.954), but was different among sample types (p<0.006), with effluent having significantly fewer fibers than sewage and sludge (p = 0.024 and 0.013, respectively). These data indicate that WWTPs selectively retained microplastic fibers in sludge. Pellets showed the opposite trend, with no significant differences between WWTPs (p = 0.246), but the relative abundance of pellets was different among sample types (p = 0.014): higher in effluent (>20%) than in sewage (<10%) and sludge (< 5%), indicating that pellets were not retained by WWTPs as effectively as fibers. There were no significant differences between WWTPs or sample types in relative abundance of other microplastic types (fragments, foam, or film).

**Fig 2:**
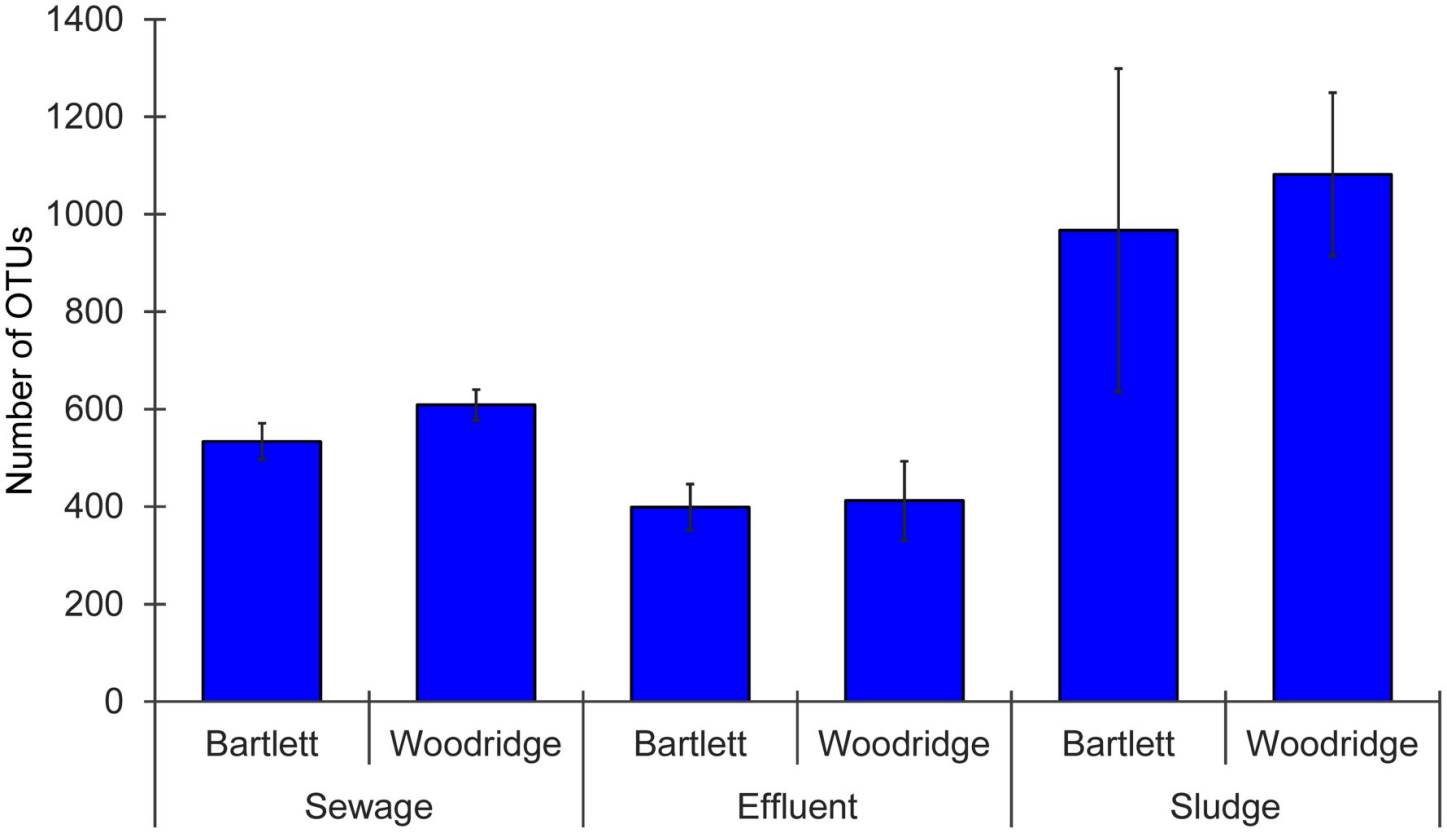
Number of operational taxonomic units (based on 97% identity in 16S rRNA amplicons) observed within bacterial communities attached to microplastic particles collected from three sample types (sewage, effluent, and sludge) from two WWTPs (Bartlett and Woodridge). Each data point represents the mean value (n = 4) with error bars representing standard error.

Bacterial assemblages attached to microplastic samples were analyzed by high-throughput amplicon sequencing of 16 rRNA genes. Bacterial assemblage taxonomic richness (i.e., the number of OTUs observed), was significantly different among sample types (sewage, effluent, and sludge) (2-way ANOVA, p = 0.001) but there was no difference between WWTPs (p = 0.547) and no significant interaction (p = 0.933) (Fig 2). Microplastics in sludge had significantly higher bacterial taxonomic richness than microplastics in sewage or effluent, which were not different from one another. In contrast to taxonomic richness, Shannon diversity of bacterial assemblages attached to microplastics showed no significant differences among sample types (2-way ANOVA, p = 0.641) or between WWTPs (p = 0.456), and no significant interaction (p = 0.701) (S2 Table).

**Fig 3:**
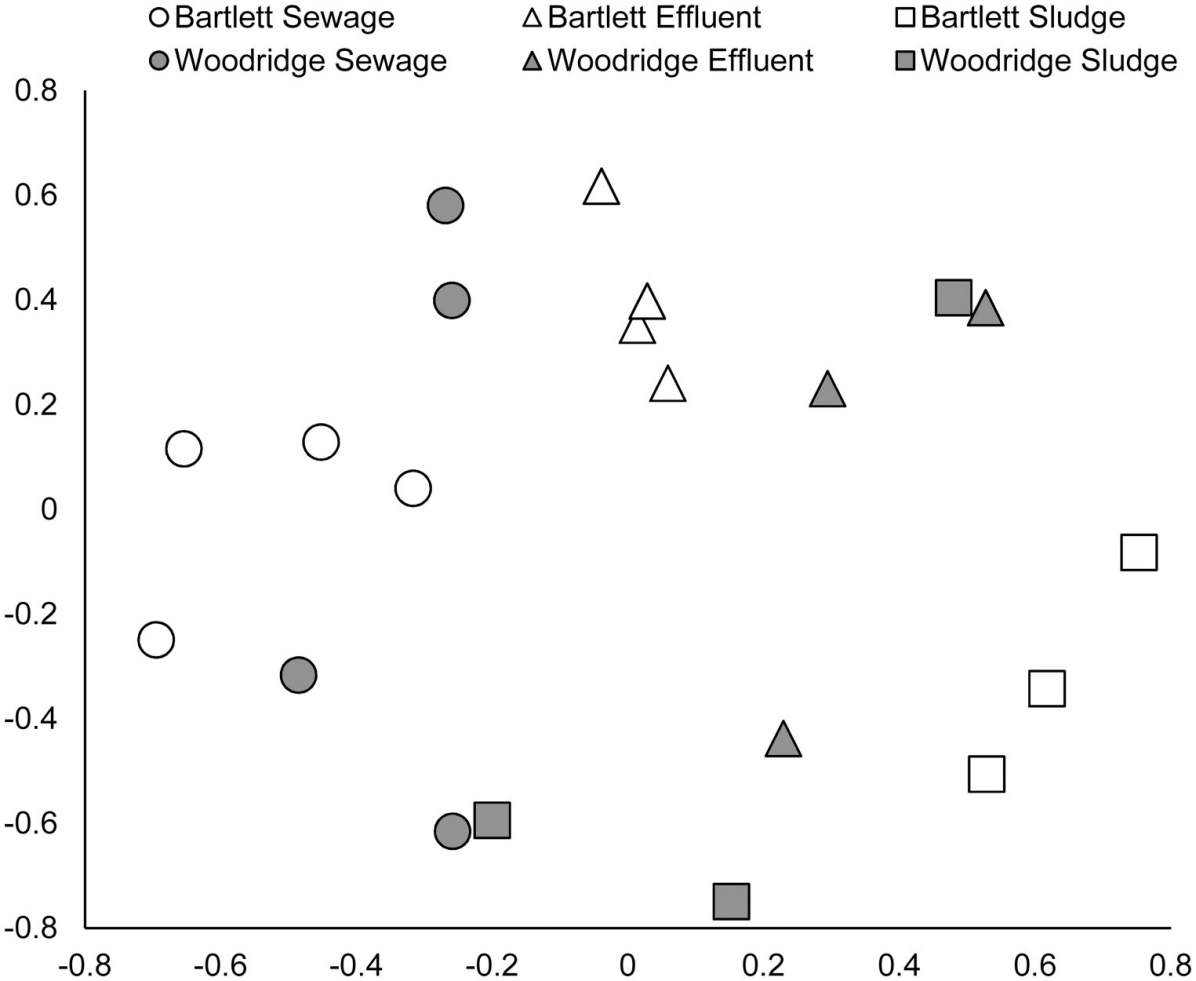
nMDS ordination of bacterial assemblage composition for biofilms attached to microplastic particles collected from three sample types (sewage, effluent, and sludge) from two WWTPs (Bartlett and Woodridge). Each point represents the bacterial assemblage from one individual sample. Bacterial assemblage analysis was based on high-throughput amplicon sequencing of partial 16 rRNA genes, clustering sequences into OTUs (97% sequence identity) and comparison of assemblages based on the theta index. Stress value of ordination = 0.2997.

Multivariate statistical analyses (nMDS ordination and AMOVA) based on OTUs (97% sequence identity) indicated no significant difference in composition of microplastic-attached bacterial assemblages from the two WWTPs (p = 0.183), but there were significant differences among the 3 sample types (p<0.001). Each sample type had a microplastic-attached bacterial assemblage distinct from the others (sewage vs. effluent p = 0.003, sewage vs. sludge p = 0.007, and effluent vs. sludge p = 0.011) (Fig 3). Pairwise comparisons of microplastic-attached bacterial assemblages from each of the sample types (sewage, effluent, and sludge) across plants showed a significant difference in bacterial assemblage between Bartlett effluent and Woodridge effluent (p = 0.027) but no significant differences in bacterial assemblage by plant for sewage (p = 0.189) or sludge (0.094). As expected, grouping of 16S rRNA gene sequences into ASVs identified a higher total number of ASVs (30,946) compared to OTUs based on 97% sequence identity (11,307), but beta diversity analyses based on ASVs produced results that were equivalent to the analyses based on OTUs. Specifically, AMOVA of the ASV data showed no significant difference in composition of microplastic-attached bacterial assemblages from the two WWTPs (p = 0.183), but there were significant differences among the 3 sample types (p<0.001) and significant pairwise differences between the sample types (sewage vs. effluent p = <0.001, sewage vs. sludge p = 0.012, and effluent vs. sludge p = 0.009). In addition, nMDS ordination based on the ASV data (S1 Fig) showed a highly similar pattern to that based on OTUs (Fig 3).

**Fig 4:**
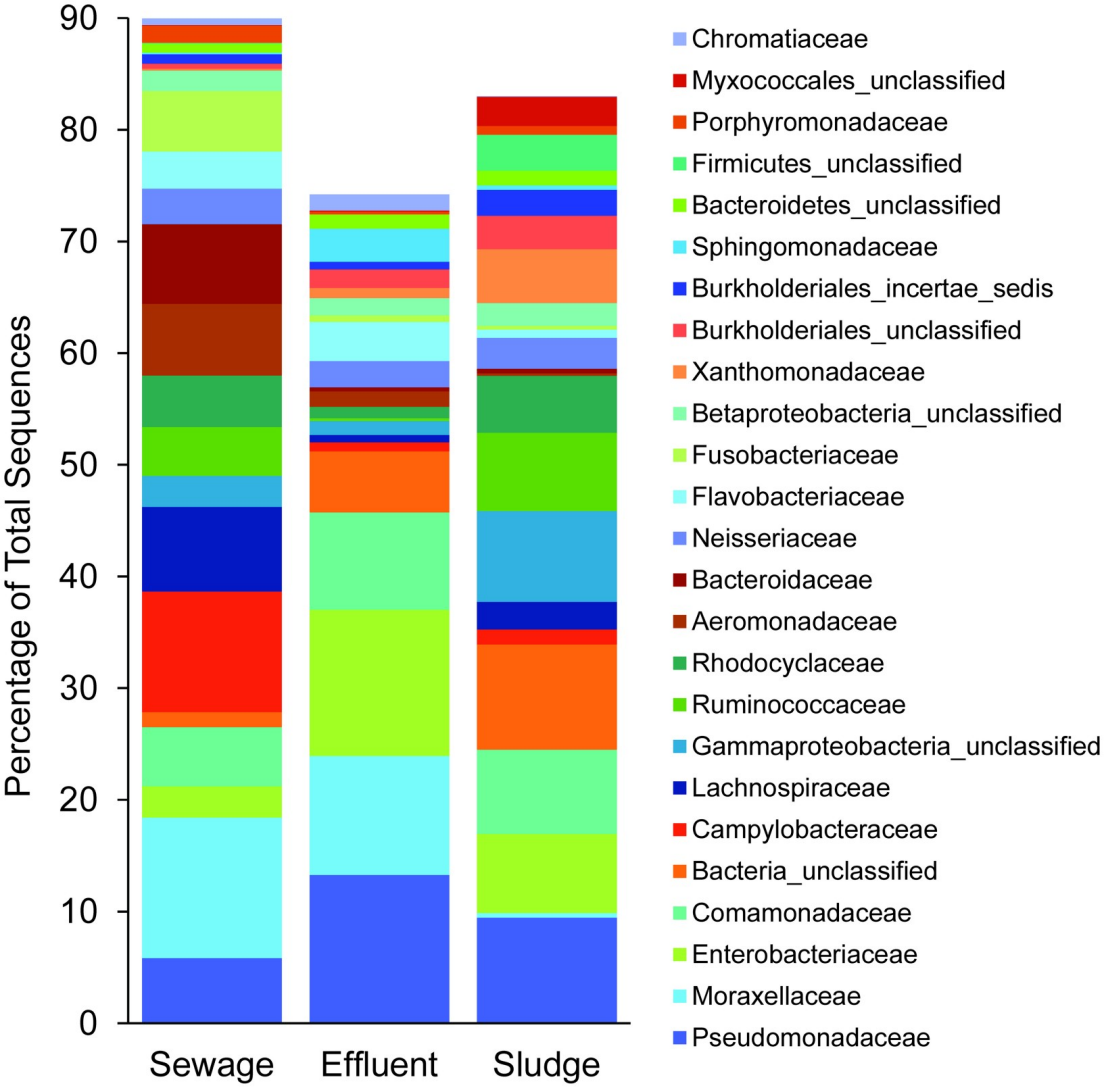
Average relative abundance of the 25 most abundant bacterial families within microplastic-attached bacterial assemblages from three sample types (sewage, effluent, and sludge). Each bar represents the mean (n = 8). Bacterial families were identified based on high-throughput amplicon sequencing of partial 16 rRNA genes.

Microplastic-attached bacterial assemblages included several families that contain pathogenic bacterial taxa or taxa associated with the human gut microbiome (Fig 4), and sequences from some of these families varied in relative abundance in different stages of wastewater treatment. For example, *Campylobacteraceae* sequence abundance varied significantly with sample type (p = 0.001), representing ~11% of bacterial sequences on sewage microplastic, but less than 1% on effluent microplastic (p = 0.002) and approximately 1% on sludge microplastic (p = 0.004). Within the family *Campylobacteraceae* 94% of the sequences were assigned to the genus *Arcobacter*. Sequences from several other families, including *Bacteroidaceae*, *Aeromonadaceae*, and *Lachnospiraceae*, showed similar trends, being most abundant on sewage microplastics and less abundant on effluent and sludge microplastics, but the effects of sample type on relative abundances of these families were not significant (p = 0.188, 0.150, 0.079, respectively). Abundance of *Moraxellaceae* sequences did not differ between sewage and effluent microplastics (~12% and 11%, respectively), but was much lower on sludge microplastic (<0.5%), although this difference was not statistically significant (p = 0.143). The genus *Acinetobacter* accounted for 96% of sequences identified to the *Moraxellaceae* family, and *Acinetobacter* sequences were similarly abundant on both sewage and effluent microplastics (p = 0.909) but significantly less abundant on sludge microplastics (p = 0.009). Finally, the relative abundance of the family *Sphingomonadaceae* varied significantly with sample type (p<0.001) and was ~10 fold more abundant on effluent microplastics than sewage or sludge microplastics. Within the family *Sphingomonadaceae* 69% of the sequences were assigned to the genus *Sphingomonas*.

Several bacterial OTUs had significant differences in relative abundance between sewage and effluent microplastics (Table 3). Relative abundance of an unclassified *Enterobacteriaceae* genus accounted for more than 11% of bacterial sequences on effluent microplastics but only ~1% on sewage microplastics. BLAST analysis indicated that the representative sequence from this unclassified genus showed highest percent identity (>98%) to several species within the genus *Klebsiella*, including *Klebsiella pneumoniae* and *Klebsiella aerogenes*. Similarly, sequences from the genus *Sphingomonas* were greater than 10-fold more abundant and sequences from the genus *Pseudomonas* were twice as abundant on effluent microplastics compared to sewage microplastic. In contrast, several OTUs were significantly less abundant on effluent microplastics, including *Arcobacter*, which decreased by a factor of 20, and an unclassified *Gammaproteobacteria* genus, which decreased by a factor of 10. The representative sequence from this unclassified *Gammaproteobacteria* genus fell within the family *Aeromonadaceae* and had a percent identity >96% for multiple species within the genus *Aeromonas*.

**Table 3:** Relative abundances of bacterial OTUs with the largest differences in relative abundance between sewage microplastic and effluent microplastic.

Genus	Sewage^1^		Effluent^1^		p-value^2^	
*Enterobacteriaceae*_unclassified	1.15	(± 0.33)%	11.03	(± 4.54)%	0.007	*
*Arcobacter*	7.38	(± 2.05)	0.34	(± 0.16)	0.000	*
*Pseudomonas*	3.72	(± 1.67)	7.68	(± 1.42)	0.022	*
*Gammaproteobacteria*_unclassified	2.35	(± 0.61)	0.29	(± 0.11)	0.000	*
*Uliginosibacterium*	0.84	(± 0.46)	0.00	(± 0.00)	0.021	*
*Aurantimonas*	0.10	(± 0.04)	0.92	(± 0.53)	0.046	
*Propionivibrio*	1.02	(± 0.23)	0.21	(± 0.12)	0.000	*
*Sphingomonas*	0.06	(± 0.04)	0.86	(± 0.30)	0.001	*
*Pantoea*	0.17	(± 0.08)	0.96	(± 0.29)	0.001	*
*Zoogloea*	0.94	(± 0.27)	0.16	(± 0.07)	0.001	*
*Rhizobium*	0.05	(± 0.02)	0.81	(± 0.34)	0.006	*
*Faecalibacterium*	0.67	(± 0.30)	0.02	(± 0.01)	0.007	*
*Roseburia*	0.70	(± 0.29)	0.08	(± 0.04)	0.008	*
*Formivibrio*	0.65	(± 0.37)	0.07	(± 0.05)	0.047	

^1^ Data represent mean values (n = 8) ± standard error.

^2^ Asterisks (*) indicate significant effects of sample type (sewage v. effluent) based on Benjamini-Hochberg correction.

Several bacterial OTUs also had significant differences in relative abundance between sewage and sludge microplastics samples (Table 4). There were significantly lower relative abundances of *Acinetobacter* (36-fold), *Arcobacter* (17-fold), *Aeromonas* (27-fold), an unclassified *Comamonadaceae* genus (37-fold), and an unclassified *Gammaproteobacteria* genus (9-fold) on sludge microplastics compared to sewage microplastics. The representative sequence from the unclassified *Comamonadaceae* genus had a percent identity >98% for multiple species within the genus *Acidovorax*, and the unclassified *Gammaproteobacteria* genus was the same OTU discussed above that matched with the genus *Aeromonas*. Sludge microplastics also had significantly higher relative abundances of another unclassified *Comamonadaceae* genus (30-fold) and an unclassified *Xanthomonadaceae* genus (100-fold). The representative sequence from this unclassified *Comamonadaceae* genus had a 99.6% identity to a strain from the genus *Ideonella*.

**Table 4:** Relative abundances of bacterial OTUs with the largest differences in relative abundance between sewage microplastic and sludge microplastic.

Genus	Sewage^1^		Sludge^1^		p-value^2^	
*Acinetobacter*	9.94	(± 4.45)%	0.28	(± 0.23)%	0.009	*
*Arcobacter*	7.38	(± 2.05)	0.43	(± 0.40)	0.000	*
*Comamonadaceae*_unclassified	0.15	(± 0.04)	5.44	(± 2.94)	0.030	*
*Aeromonas*	5.02	(± 2.45)	0.18	(± 0.11)	0.017	*
*Comamonadaceae*_unclassified	3.21	(± 1.66)	0.35	(± 0.09)	0.037	*
*Gammaproteobacteria*_unclassified	2.35	(± 0.61)	0.26	(± 0.25)	0.001	*
*Flavobacterium*	1.72	(± 0.88)	0.14	(± 0.13)	0.033	*
*Arcobacter*	1.94	(± 0.40)	0.57	(± 0.41)	0.004	*
*Xanthomonadaceae*_unclassified	0.01	(± 0.01)	1.00	(± 0.49)	0.015	*
*Zoogloea*	0.94	(± 0.27)	0.14	(± 0.10)	0.002	*
*Propionivibrio*	1.02	(± 0.23)	0.28	(± 0.24)	0.008	*
*Trichococcus*	0.64	(± 0.29)	0.05	(± 0.03)	0.015	*
*Cloacibacterium*	0.67	(± 0.16)	0.13	(± 0.08)	0.001	*
*Bacteroides*	0.52	(± 0.24)	0.06	(± 0.05)	0.021	*
*Sulfurospirillum*	0.57	(± 0.19)	0.10	(± 0.06)	0.005	*

^1^ Data represent mean values (n = 8) ± standard error.

^2^ Asterisks (*) indicate significant effects of sample type (sewage v. sludge) based on Benjamini-Hochberg correction.

## Discussion

Microplastics were identified in untreated sewage, effluent water, and sludge from two WWTPs treating primarily domestic wastewater. Patterns of particle abundance throughout the WWTPs were consistent with previous assessments. Microplastic concentrations in untreated sewage ranged from 800–2,000 particles/m^3^, which is comparable to published data which ranged from 1,000 to > 50,000 particles/m^3^ [24–26]. Both WWTPs in this study retained > 99% of influent microplastic, which is similar to [24, 25, 27] or higher than [26, 64] values reported in prior studies, which ranged from 72% to 99%. The slight difference in retention between the two WWTPs suggests that sand filtration of effluent enhanced microplastic retention, which has been reported previously [8, 65]. Effluent microplastic concentrations ≤1 particle/m^3^, which is on the low end of published values (for review see [66]), support the conclusion that both WWTPs were effective at removal of microplastics. Despite high retention both WWTPs released microplastic in their effluent on the order of hundreds to thousands of pieces per day. This finding agrees with a previous study which reported that both of these WWTPs were point sources of microplastics to their receiving streams [8] and with studies that have detected microplastics in effluent from a range of WWTPs [28, 29]. However, the very low concentration of microplastic in the effluent from both plants suggests the possibility that daily microplastic discharge could be highly variable, so future studies should examine temporal variability in effluent microplastic concentrations.

Changes in the relative abundance of microplastic particle types between sewage and effluent suggest selective retention. Microfibers were the most common type of microplastics in influent sewage (>50%) and showed higher relative abundance in the sludge relative to the effluent, suggesting net fiber retention. While similar selective retention of fibers was reported in previous studies [25, 67], fibers are one of the most common forms of microplastics detected in aquatic habitats [8, 23, 31]. One possible explanation for this discrepancy is that fibers may be entering aquatic ecosystems through additional routes, including surface runoff, airborne deposition, or direct release of untreated wastewater via combined sewer overflows or leaking sewage infrastructure [31, 68]. In contrast to fibers, pellets were less well retained by WWTPs and increased in relative abundance in effluent water. Previous studies have also reported that pellets are less well retained by WWTPs compared to other microplastic types [26]. The contrasting retention of fibers and pellets may reflect differences in their physical properties, such as density, shape, buoyancy, or hydrophobicity, that might influence the tendency of these particles to settle or become trapped within flocs. Determination of properties that influence microplastic retention in WWTPs would be a good topic for future controlled, manipulative experiments.

High retention of microplastic particles by both WWTPs resulted in high concentrations of microplastics in sludge, similar to previous reports [67]. Concentrations measured in this study for untreated sludge were on the order of hundreds of thousands of particles/m^3^, which is approximately ten times lower than reported in a previous study of digested sludge [25], with this discrepancy likely due to the reduction in sludge volume resulting from digestion which would lead to increased microplastic concentration. Sewage sludge is commonly applied to land in the U.S. [69], and there is no regulatory framework to assess or limit sludge microplastics [70]. Thus, microplastics in sludge may be a concern for soil ecosystems [71]. In addition, surface runoff from applied biosolids could transport land-applied microplastic to surface waters (i.e., agricultural streams), but this has not yet been assessed.

Microplastics in the environment encompass a wide diversity of sizes and materials [72], some of which were not captured with our methods. Microplastics smaller than 300 microns (including particles in the nanometer size range) were not included in the sampling techniques used in our study, suggesting that our numbers may be an underestimate of the total microplastics in wastewater. Furthermore, microplastics with a density > 1.3 g cm^-3^ (e.g. polyvinyl chloride) were less likely to be captured with the salinity-based separation we used. This would be especially relevant for the sludge, since high density particles would be most likely to have settled into the sludge. Additional work is needed to consolidate an array of sampling methods that could capture a broader range of the total microplastic particle assemblage in wastewater.

Microplastic particles from all samples were colonized by diverse microbial assemblages, which were attached to the plastic surfaces as they were not washed off during microplastic collection. Several of the most abundant taxa within these microplastic-attached bacterial assemblages are known biofilm formers, including *Pseudomonadaceae* [73], *Moraxellaceae* [74], *Enterobacteriaceae* [75], and *Comamonadaceae* [76]. Our study represents the first analysis of microplastic-attached bacterial assemblages within WWTPs and our results suggest that the wastewater treatment process has strong effects on these assemblages. Species richness and taxonomic composition differed across stages of wastewater treatment, and trends were consistent for the two WWTPs included in this study. Specifically, there was no difference in species richness of bacterial assemblages attached to microplastic in influent sewage versus effluent water, but there was a significant difference in taxonomic composition of these assemblages.

Several potentially pathogenic bacterial taxa were lower in abundance on effluent microplastics than on influent sewage microplastics, suggesting that these taxa were negatively affected by passage of microplastic through WWTPs. For example, sequences identified to the genus *Arcobacter* and to its family *Campylobacteraceae* were significantly more abundant on microplastics in influent sewage and less on effluent and sludge microplastic. The family *Campylobacteraceae* and the genus *Arcobacter* include multiple taxa associated with human gastrointestinal infections such as gastroenteritis [77, 78]. The same pattern was observed for the genus *Aeromonas*, which includes multiple species associated with human disease [79]. The decreases in relative abundances of *Campylobacteraceae*, *Arcobacter*, and *Aeromonas* on effluent microplastics suggests that wastewater treatment could help to limit release of these potentially pathogenic taxa to the environment. However, previous studies have identified these taxa on microplastics in rivers receiving wastewater inputs [6, 32], including the rivers receiving effluent from these specific WWTPs [8], so wastewater treatment may not completely remove these taxa from microplastics.

Other bacterial taxa associated with human infections were abundant on microplastics in both influent sewage and effluent water, suggesting that abundance of these taxa was not altered by wastewater treatment. For example, the genus *Acinetobacter* and its family *Moraxellaceae* were abundant on both sewage and effluent microplastics. Members of the genus *Acinetobacter* are involved in a wide range of human infectious diseases and are a common cause of nosocomial infections [80]. Failure of wastewater treatment to reduce the abundance of these taxa on microplastics could result in their release to the environment, and indeed these taxa were identified previously on microplastics in rivers receiving wastewater inputs [6, 32], including the rivers receiving effluent from the specific WWTPs analyzed in this study [8]. Moreover, prior work demonstrated that *Moraxellaceae* sequences were still detected on microplastics up to 2km downstream of a WWTP [32], suggesting that microplastics could be a vector for transport of this potentially pathogenic taxon within rivers.

Finally, sequences from several bacterial genera were significantly more abundant on effluent microplastics compared to sewage microplastic, suggesting that these taxa increased in abundance during wastewater treatment. Sequences from the family *Enterobacteriaceae* that had a high percentage identity to *Klebsiella pneumoniae* and *Klebsiella aerogenes* increased by a factor of 10. Both of these *Klebsiella* species are part of the normal human microbiome, but they are also common causes of opportunistic and nosocomial infections [81]. *Klebsiella pneumoniae* has also been shown to be capable of biodegrading polyethylene [82]. We are not aware of previous studies reporting the presence of *Klebsiella* on microplastics, but it’s high abundance on effluent microplastics is noteworthy due to its connections to humans and to plastic breakdown. Sequences from the *Pseudomonas* genus were also significantly more abundant on effluent microplastics compared to sewage microplastics. Species from the genus *Pseudomonas* are common biofilm formers [83] and have been linked to breakdown of a wide range of plastic polymers [84–89] as well as production of enzymes that contribute to plastic biodegradation [90]. The abundance of *Pseudomonas* sequences on microplastics within WWTP effluent agrees with previous detection of this genus on microplastic collected from urban rivers, including the rivers receiving effluent from the WWTPs analyzed in this study [6, 8, 32]. Moreover, prior work demonstrated that *Pseudomonas* increased in abundance on microplastics in an urban river with distance from a WWTP [32]. Finally, sequences from the genus *Sphingomonas* and its family *Sphingomonadaceae* were 10-fold more abundant on effluent microplastic compared to sewage microplastic. Bacteria from the genus *Sphingomonas* are able to degrade various complex organic compounds, including polycyclic aromatic hydrocarbons [91], the plasticizer bisphenol A [92] and plastic monomers [93, 94], and they produce a polysaccharide that enhances biofilm formation on plastic surfaces [95]. The observed increases in *Klebsiella*, *Pseudomonas*, and *Sphingomonas* on microplastics in effluent suggests some specific selection for these organisms on plastic within WWTPs, perhaps related to the surface as a growth substrate and their capacity to break down plastic polymers.

The observed shifts in the composition of microplastic bacterial assemblages during passage through the WWTPs could be driven by several factors, including interactions with microbes within the plants and changes in environmental conditions at different stages of treatment. For example, oxygen concentration can affect biofilm development [96] and the active pumping of air into the aeration tank increases oxygen availability. Experimental investigations of the effects of oxygen on microplastic biofilm development could provide insights into the mechanisms underlying the observed shifts in microplastic bacterial assemblages during wastewater treatment.

Bacterial assemblages attached to microplastics in sludge were also different than influent sewage, suggesting that microplastic-attached bacterial assemblages changed during transfer of microplastics from sewage to sludge. Bacterial assemblages on sludge microplastics showed higher species richness, indicating that additional bacterial taxa colonized microplastics during wastewater treatment. This colonization may enhance plastic retention, perhaps by increasing sedimentation rates within the settling tank. Previous work has demonstrated that microbial colonization of microplastics can increase settling of microplastic particles [43, 44] and microplastic retention in streams [46]. Bacterial colonization of microplastics during wastewater treatment could also enhance retention by incorporating microplastics into flocs. In our study, sequences from the family *Xanthomonadaceae* and one unclassified *Xanthomonadaceae* genus were dramatically more abundant on sludge microplastics than sewage microplastics. A *Xanthomonas* strain was previously isolated from sewage sludge and was shown to have high surface hydrophobicity and ability to co-aggregate with other wastewater bacteria, suggesting that it might play a role in bioflocculation within WWTPs [97]. Microplastic surfaces are also hydrophobic, which might enhance interaction with *Xanthomonas* and contribute to incorporation of microplastics into flocs. Sequences from an unclassified *Comamonadaceae* genus that matched to the genus *Ideonella* were also dramatically more abundant on sludge microplastic than sewage microplastic. An *Ideonella* species was recently reported to be capable of degrading and assimilating carbon from polyethylene [98], so the increase in this taxa in sludge may be linked to plastic breakdown. The potential for WWTP bacteria to biodegrade microplastics would be a good topic for future controlled, manipulative experiments. In addition, the composition of microbial communities within WWTPs can vary seasonally [99, 100] and by geographic location [101, 102], so analyzing microplastic microbiomes within WWTPs across seasons and in more spatially separated locations would be valuable.

## Supporting information

S1 Fig nMDS ordination of bacterial assemblage composition for biofilms attached to microplastic particles collected from three sample types (sewage, effluent, and sludge) from two WWTPs (Bartlett and Woodridge). Each point represents the bacterial assemblage from one individual sample. Bacterial assemblage analysis was based on high-throughput amplicon sequencing of partial 16 rRNA genes, grouping sequences into ASVs, and comparison of assemblages based on the theta index. Stress value of ordination = 0.2997.
(PDF)

S1 Table Identification of microplastic polymer types by PyGCMS. (PDF)

S2 Table Shannon diversity of microplastic attached bacterial assemblages. (PDF)
