# Supplementary material for: Wastewater treatment alters microbial colonization of microplastics
Source: PLoS One. 2021 Jan 6;16(1):e0244443. doi: 10.1371/journal.pone.0244443 (PMC7787475; doi:10.1371/journal.pone.0244443)
Supplement: S1 Fig — Each point represents the bacterial assemblage from one individual sample. Bacterial assemblage analysis was based on high-throughput amplicon sequencing of partial 16 rRNA genes, grouping sequences into ASVs, and comparison of assemblages based on the theta index. Stress value of ordination = 0.2997. (PDF) [file pone.0244443.s001.pdf]

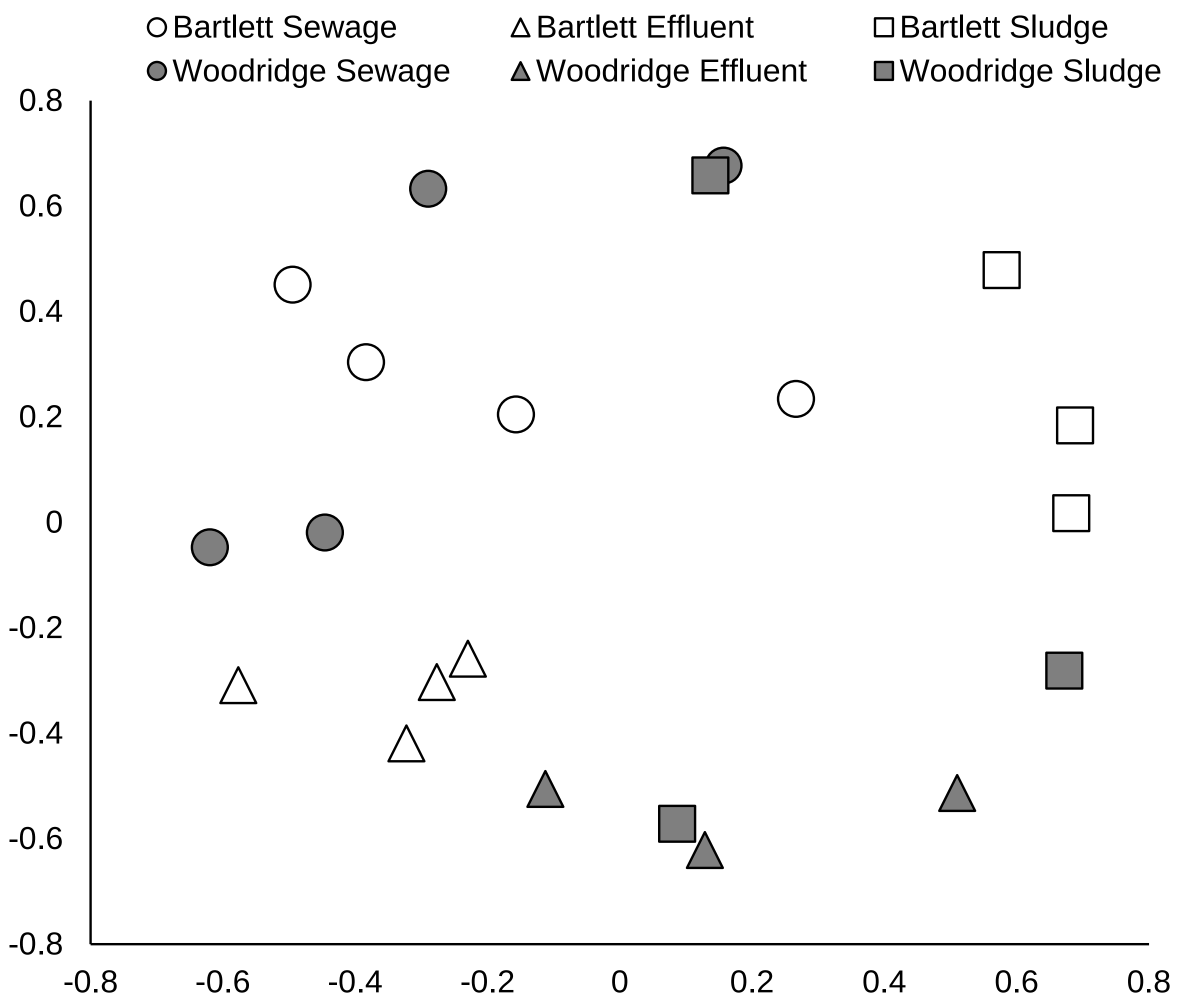

S1 Figure. nMDS ordination of bacterial assemblage composition for biofilms attached to microplastic particles collected from three sample types (sewage, effluent, and sludge) from two WWTPs (Bartlett and Woodridge). Each point represents the bacterial assemblage from one individual sample. Bacterial assemblage analysis was based on high-throughput amplicon sequencing of partial 16 rRNA genes, grouping sequences into ASVs, and comparison of assemblages based on the theta index. Stress value of ordination = 0.2997.
