# Supplementary material for: Wastewater treatment alters microbial colonization of microplastics
Source: PLoS One. 2021 Jan 6;16(1):e0244443. doi: 10.1371/journal.pone.0244443 (PMC7787475; doi:10.1371/journal.pone.0244443)
Supplement: S1 Table — (PDF) [file pone.0244443.s002.pdf]

S1 Table. Identification of polymer types by PyGCMS

Sample	Polymer Type
Bartlett Foam	Polyethylene
Bartlett Fibers	Polyethylene
Bartlett Pellet	Epoxy Resin
Woodridge Fragment	Polypropylene
Woodridge Foam	Polystyrene
Woodridge Fiber	Polypropylene
Woodridge Pellet	Not Identified
