# Supplementary material for: Wastewater treatment alters microbial colonization of microplastics
Source: PLoS One. 2021 Jan 6;16(1):e0244443. doi: 10.1371/journal.pone.0244443 (PMC7787475; doi:10.1371/journal.pone.0244443)
Supplement: S2 Table — (PDF) [file pone.0244443.s003.pdf]

S2 Table. Shannon diversity of microplastic-attached bacterial assemblages.

Plant	Sewage[1]	Effluent[1]	Sludge[1]
Bartlett	4.09 (± 0.22)	4.46 (± 0.30)	3.94 (± 0.77)
Woodridge	3.70 (± 0.21)	4.01 (± 0.38)	4.09 (± 0.27)

[1] Data represent mean values (n=4) ± standard error.
